# A platform for leveraging next generation sequencing for routine microbiology and public health use

**DOI:** 10.1186/2047-2501-3-S1-S7

**Published:** 2015-02-24

**Authors:** Laura I Rusu, Kelly L Wyres, Matthias Reumann, Carlos Queiroz, Alexe Bojovschi, Tom Conway, Saurabh Garg, David J Edwards, Geoff Hogg, Kathryn E Holt

**Affiliations:** 1 IBM Research, Australia, 204 Lygon Street, Carlton, Victoria 3053, Australia; 2 Department of Biochemistry and Molecular Biology and Bio21 MolecularScience and Biotechnology Institute, The University of Melbourne, Parkville VIC 3010, Victoria, Australia; 3 Microbiological Diagnostic Unit Public Health Laboratory, The University of Melbourne, Parkville Victoria 3010, Australia

## Abstract

Even with the advent of next-generation sequencing (NGS) technologies which have revolutionised the field of bacterial genomics in recent years, a major barrier still exists to the implementation of NGS for *routine *microbiological use (in public health and clinical microbiology laboratories). Such routine use would make a big difference to investigations of pathogen transmission and prevention/control of (sometimes lethal) infections.

The inherent complexity and high frequency of data analyses on very large sets of bacterial DNA sequence data, the ability to ensure data provenance and automatically track and log all analyses for audit purposes, the need for quick and accurate results, together with an essential user-friendly interface for regular *non-technical *laboratory staff, are all critical requirements for routine use in a public health setting. There are currently no systems to answer positively to all these requirements, in an integrated manner.

In this paper, we describe a system for sequence analysis and interpretation that is highly automated and tackles the issues raised earlier, and that is designed for use in diagnostic laboratories by healthcare workers with no specialist bioinformatics knowledge.

## Introduction

Despite the development of effective antimicrobial drugs and anti-bacterial vaccinations, pathogenic bacteria continue to cause significant human morbidity and mortality in all regions of the world [1-4]. The situation is exacerbated by the evolution and spread of antibiotic resistance, a phenomenon identified as a major global health issue by the World Health Organization [5]. In order to reduce the burden of bacterial disease it is imperative that infections are diagnosed and characterised in a timely manner, and that the evolutionary and epidemiological dynamics of bacterial populations are investigated. Microbiology diagnostic and public health laboratories play a primary role in such tasks, and in this context bacterial genome sequence analysis holds immense transformative potential [6,7].

Genomic sequences can be generated through the use of next-generation sequencing (NGS) technologies, which have been rapidly advancing in recent years. The application of these technologies is revolutionising diagnostic and public health microbiology due to the high yield and resolution, low turn-around time and falling costs of the data obtained [6,7]. Indeed there are already case studies that demonstrate the feasibility of using NGS in this context e.g. for high resolution investigation of hospital acquired bacterial disease outbreaks or rapid estimation of bacterial antimicrobial susceptibility [8,9]. In such cases information derived from NGS data can rapidly inform clinical and public health decisions, thereby improving infection control and patient outcomes.

The challenge in making NGS a reality for routine diagnostic and public health laboratory use is that the analysis is far from trivial, comprising multiple steps (e.g. mapping or assembly of the genome, variant calling and comparative phylogenetic analyses) [10]. Each step can be implemented using different tools and algorithms, which may require case-by-case optimisation. This process is time consuming and cumbersome since most of the tools work in isolation and do not have a user-friendly interface. Simple tools that would allow healthcare professionals and laboratory staff to carry out the analyses are not widely available, thus limiting the analyses only to those with bioinformatics expertise [7,8,11]. Further, the volume and variety of bacterial genomes to be analysed present organisational and data management challenges.

### Next generation sequencing for microbiological diagnostics

Microbiology laboratories may process hundreds of bacterial isolates each week [6]. Clinical laboratories receive patient samples from which clinically-relevant bacteria are cultured, purified and identified to an appropriate taxonomic level [6,7]. In contrast, specialised public health or reference laboratories receive pre-purified bacterial stocks. The scale and variety of bacterial isolates encountered by such laboratories is exemplified by the microbiology laboratory at the Oxford University Hospitals Trust, UK, where 751,134 isolates were cultured over a 15 year period (i.e. approximately 960 isolates per week). Seventy-four percent of these isolates were classified to the species level and represented a total of 301 different species [6].

Identification may be followed by further characterisation, whereby the required spectrum of tests is dependent upon the bacterial species or clinical situation in question. Such tests, for which the specific protocols also vary in a species-dependent manner, include antibiotic susceptibility testing, epidemiological typing, toxin and virulence gene screening. The necessary laboratory techniques may include microscopy, susceptibility testing by disc diffusion or E-test, biochemical assay, polymerase chain reaction and capilliary sequencing. As such, full bacterial characterisation may require several days or even weeks for completion [6,7].

In contrast to traditional methods, NGS data representing almost an entire bacterial genome can be generated in a matter of hours, independent of species classification [12,13]. These data can provide much of the information required for bacterial characterisation and/or comparison, including epidemiological typing [14,15], antibiotic-resistance, toxin and virulence gene information [14,16,17]. Furthermore, NGS data provide information at a much higher resolution than that of traditional techniques, allowing fine-scale epidemiological investigations. Such enhanced resolution has already been shown to facilitate improved detection of pathogen transmission and outbreak foci [8,18,19]. In particular, an investigation of a set of meticillin-resistant *Staphylococcus aureus *infections in a British hospital identified a staff member as the focal point of transmission and led to the formulation of an appropriate intervention strategy. Crucially, it was shown that these findings could not be determined by the use of traditional characterisation techniques alone [8].

Unfortunately given that microbiology laboratory staff should not be expected to possess advanced bioinformatics skills, the lack of automated and user-friendly NGS analysis tools is a major barrier to the routine use of NGS technologies in public health and diagnostic laboratories [7,8]. There are also difficulties relating to the management of large/variable data sets and analysis record-keeping, both of which are essential if meaningful, accurate and reliable conclusions are to be drawn from the data.

### Related works

Over recent years, there have been several commercial and open source initiatives that integrate specific NGS analysis components (e.g. Pipeline Pilot [20], Taverna [21] and Galaxy [22]). These systems provide interfaces to create and run analysis pipelines using different NGS analysis tools (e.g. BWA [23], SamTools [24] and MAUVE [25]). Some of the benefits offered by these systems are accessibility (providing a user interface for running and creating NGS analysis pipelines), reproducibility (one can save the analysis and metadata associated with it), transparency (one can share and publish analysis pipelines), and the ability to integrate new tools. Most of these features satisfy the needs of a researcher with bioinformatics skills. However, they are not sufficient for routine use within microbiological diagnostics and public health laboratories. In particular, these systems lack enterprise setting capabilities, such as data management of large and variable datasets, auditing, governance and access control/security functionalities sufficient for multiple users with different responsibilities. As such these systems have not been employed in the laboratory setting.

In this paper, we propose a sequence analysis and interpretation system which is highly automated and that tackles the issues raised earlier. It is designed for use in diagnostic laboratories by healthcare workers with no specialist IT or bioinformatics knowledge.

The next section provides a high level description of the proposed system, followed by the description of a use case showing how the system would respond to a number of routine NGS analysis tasks performed by a biologist employed in a generic microbiology laboratory.

## Proposed system description

**Figure 1:**
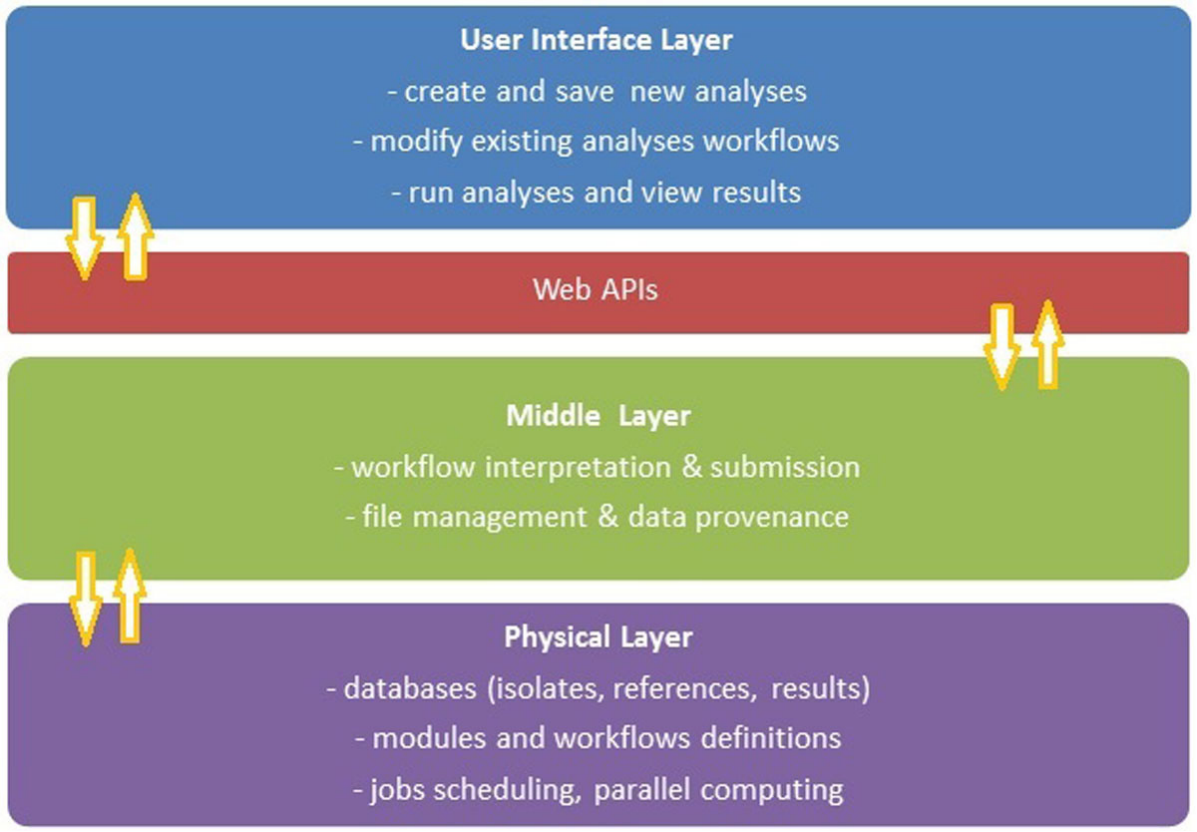
**Proposed analysis framework for whole genome sequencing analysis in diagnostic microbiology**.

The architectural framework of the system consists of four layers (illustrated in Figure 1) where the focus is on usability and where the computation is completely hidden from users, i.e. users do not need to specify or understand what computational resources should be used to carry out the analyses.

The ***User Interface (UI) Layer ***allows the user to upload data as required (sequence data files, isolate metadata, reference data etc.) and perform various searches. The user is also able to create, modify and run analyses by specifying the required input data and applicable parameters. The tool also allows creation and management of NGS analysis workflows in a visual (drag and drop) manner. A user can connect various analysis modules using arrows, thereby creating the analysis workflow. In addition to analysis modules (e.g. BWA), a workflow can include visualisation modules. If required, the user has the ability to run the same analysis workflow with different parameter sets, in parallel (e.g. to compare results), or run totally different analysis workflows at the same time. Reports can also be produced to detail or visualise results of any given analysis.

The ***Web APIs ***(Application Programing Interfaces) layer is the link between the UI and the Middle Layer. For example, once the user sets up and starts an analysis, the applicable workflow description and requirements are passed to the Middle Layer via an API. By having a standard set of APIs that are exchanged in a standard format [27], the actual implementation of the UI (e.g. web client, phone application) is independent from any specific implementation of the Middle Layer.

The ***Middle Layer ***provides a runtime environment to execute the analysis workflows. Each request received from the UI layer (e.g. a search for isolates, a new analysis run etc.) is coordinated by the Middle Layer and passed to the Physical Layer as required. For example, when a new analysis workflow definition is received from the UI, it is loaded and interpreted by the Middle Layer, and the required analysis modules are passed to the Physical Layer to be scheduled and executed in the appropriate order. If requested in the workflow definition, the Middle Layer also facilitates passing of the results for each module run, from the Physical Layer back to the UI Layer, to be visualised. The Middle Layer also ensures a solid file management and data provenance practice, by linking the information about each module of an analysis, the associated input files, parameters and results with any intermediate or output files.

The ***Physical Layer ***has two major roles: one is to store all the relevant data in the system (e.g. isolate metadata, sequence data files, reference metadata/files), together with workflow definitions and analysis results, in a database which is linked to a file management system. The other major role is to take care of scheduling and running the requests received from the Middle Layer. The Physical Layer also assesses the computational requirements for each request and allocates resources for parallel computing if required for a particular task (e.g. allocate a very intensive analysis task to be run on a computer cluster (local or remote) or a supercomputer).

### Use case example

**Figure 2:**
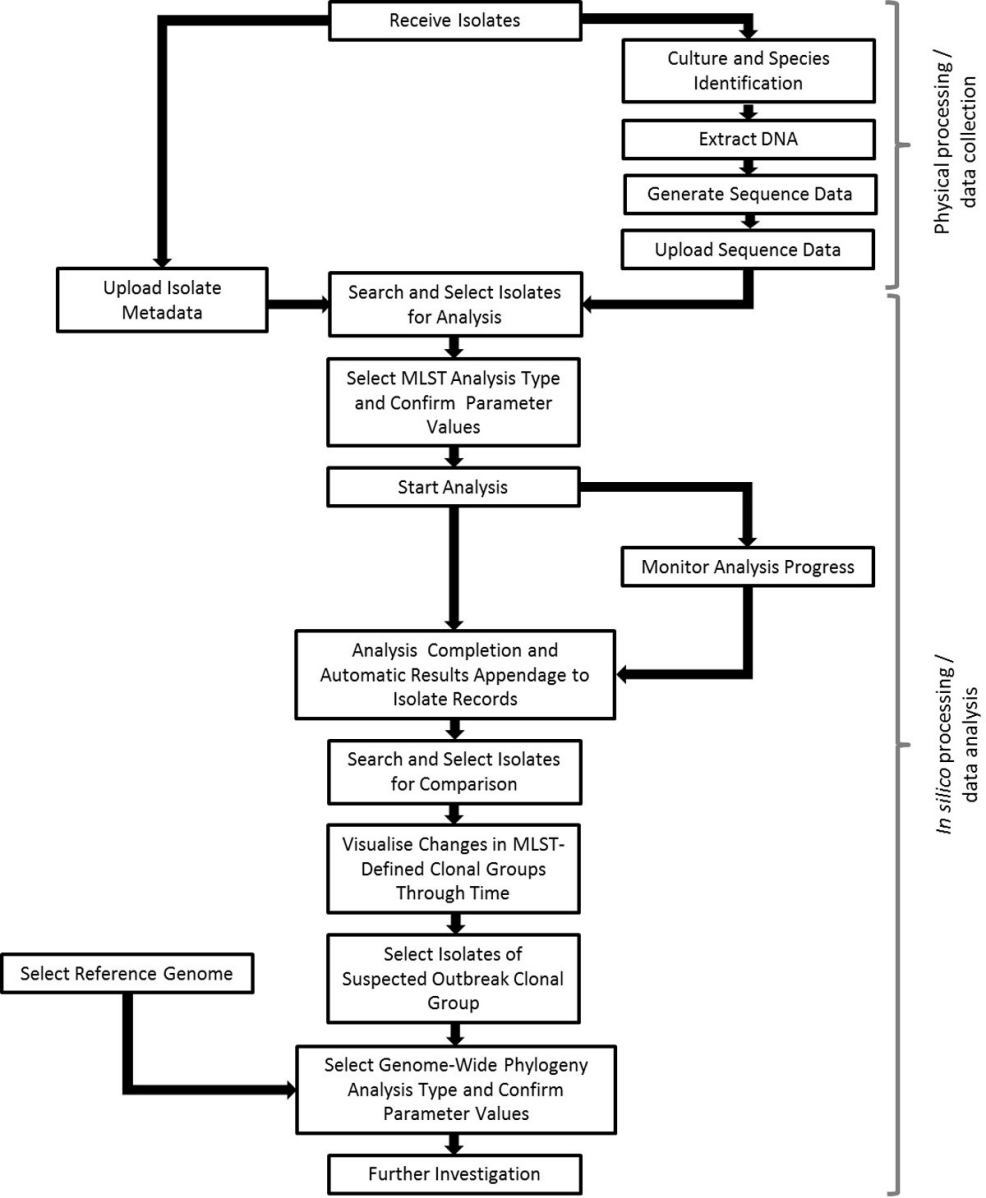
**Diagrammatic representation of the hypothetical workflow described in the use case**.

Here we describe a hypothetical analysis flow which could be performed by a biologist in a generic microbiology laboratory on a regular basis (see Figure 2 for a visual representation of the hypothetical analysis flow). For each major step in the flow we show how the user interacts with the system and what actions would be taken in order to fulfil the analysis requirements:

The microbiology laboratory has received a set of *Listeria monocytogenes *isolates and a set of *Salmonella enterica *serovar Typhi isolates for characterisation. The isolates have been cultured, their DNA extracted and sequenced on the Illumina MiSeq platform. The laboratory protocols dictate that a multi-locus sequence typing (MLST [28]) analysis should be completed for all isolates, whilst an antibiotic resistance gene analysis should be completed for the *S. enterica *ser. Typhi isolates only. Information about the isolates (including the laboratory isolate identifier, species, date of collection and date of receipt by the diagnostic laboratory) is available in a spreadsheet and uploaded into the system by a laboratory staff member.

***System access***: Upload Isolate Data;

***User Actions***: Select one or more isolate data file; upload and review success or errors; edit records and re-upload as needed;

The corresponding sequence data files (in Fastq format) are also uploaded and automatically linked by the system to the correct isolate records.

***System access: ***Upload Sequence Data;

***User actions: ***Select a folder or a subset of Fastq files; provide sequencing run details; validate the automated system matching of sequence files with the isolate data;

Firstly, the laboratory staff member would like to conduct the MLST analyses; he/she can use the isolate search functionality to identify all *L. monocytogenes *isolates in the isolate database for which an MLST analysis has not yet been completed. The "Select All" feature can be used to select all of the identified isolates for inclusion in an MLST analysis run.

***System access: ***Search Isolates;

***User actions: ***Specify search criteria as needed; select one, some or all isolate records;

The staff member can then navigate to the "Create New Analysis" screen, select the MLST analysis type and confirm the parameter values to be used (the appropriate values are set as default, see below for more details). The appropriate species-specific MLST reference database is automatically selected by the system based upon the species designation of the nominated isolates. The raw sequence data files upon which the analysis is to be performed are also selected automatically by the system, using the isolate metadata - sequence data link information. Thus there is no need for the laboratory staff member to manually search for and identify isolates to be included in the analysis, nor is there a need for the staff member to manually locate, copy or move the appropriate raw sequence data files. The laboratory staff member can start the MLST analysis simply by clicking the "Start" button. There is no need for the staff member to select a specific analysis algorithm since the system stores and utilises a list of default 'preferred' algorithms (see below for more details, e.g. SRST [29] for MLST analysis). There is no need for the staff member to allocate specific compute resources or provide information about the memory or wall-time requirements for the analysis job. The system will handle all such requirements and make appropriate decisions.

***System access: ***Create New Analysis;

***User actions: ***Specify species of isolates; specify type of analysis; select isolate records to be used; specify reference file; specify other parameters as applicable; start analysis;

Whilst the *L. monocytogenes *analysis is running, the staff member can also set-up and start both an MLST analysis and an antibiotic resistance gene analysis for the *S. enterica *ser. Typhi isolates. The staff member can use the list of recent analyses or the analysis search tool to monitor the progress of their analyses.

***System access: ***Open most recent analyses or Search Analyses;

***User actions: ***Specify search criteria; select one, some or all analyses; view analyses details & status; stop/start analyses; pause/resume analyses;

Upon completion, the results of the analyses are automatically linked to the appropriate isolate metadata records in the isolate database. The staff member would like to compare the results of their *L. monocytogenes *MLST analysis with those completed for all *L. monocytogenes *isolates received by the laboratory within the last year; he/she can use the isolate search feature to find all of the relevant isolate metadata records, and subsequently view changes in the distribution and frequency of clonal group (groups of ancestrally closely related isolates defined by MLST data comparisons) representatives through time.

***System access: ***Search Isolates;

***User actions: ***Specify search criteria as needed; select one, some or all isolate records; view and compare analyses results;

This comparison indicates that there has been a recent increase in the number of isolates representing ST13 (multi-locus sequence type 13) and its closely related variants. Such a change in the frequency and distribution of clonal groups may indicate a local *L. monocytogenes *outbreak. In order to confirm the relatedness of the suspected outbreak isolates in comparison to other *L. monocytogenes *isolates representing the same or closely related STs, the staff member can use the isolate search feature to identify and select all closely related *L. monocytogenes *isolates for which sequence data is available.

The genomes of these isolates can be further investigated, e.g. by construction of a phylogeny through reference-based sequence mapping and variant calling across the wider *L. monocytogenes *genome.

Unlike the MLST analysis, which can be completed by the use of a single tool, this type of analysis requires a multi-module process including read-mapping to a reference sequence (e.g. using BWA [23]), variant calling (e.g. using SAMTools [24]), phylogeny construction (e.g. using RAxML [30]) and quality filtering (e.g. to trim or filter sequence reads below a quality threshold, or to apply a minimum read mapping depth, percentage read concordance and/or minimum base quality threshold for variant calls). However, the laboratory staff member is able to select and use such workflows in the single step. In this case the staff member will simply need to identify and select the isolates for inclusion and an appropriate reference genome from within the reference database, plus confirm the analysis parameters (e.g. use the default specification or an alternate choice). For example, the staff member may wish to change the minimum read depth or base quality for variant calls.

As above, the staff member does not need to think about the specific analysis tools that are required, since the default 'preferred' tools will be automatically selected by the system. Such tool (and parameter) preferences can be customised by the system administrator based on the current best-practice recommendations and laboratory preferences. However, users with the right knowledge and inclination do have the option to select alternative analysis tools for one or all of the analysis modules in the workflow.

***System access: ***Search References;

***User actions: ***Specify search criteria as needed; select the applicable reference files;

As it can be seen in the above example, the actions which a non-bioinformatics laboratory user must take in order to fulfil his/her analysis task requirements using the system are quite simple. The system automates as much of the process as possible thereby reducing manual interaction of the user with the data and the potential impact of human error. For example, the sequence data is automatically linked to the correct isolate metadata records, the appropriate species-specific reference file and the raw data files are selected automatically by the system when the user creates a new analysis run. Additionally, the results of the analyses are automatically linked to the appropriate isolate metadata records and analysis record details. Moreover, all computation related information is completely hidden from the user (e.g. the specific modules that were run, in which order, on which computer/cluster, how much memory and compute resources were required, etc.).

## Conclusions

Our aim is to facilitate the routine use of NGS technologies in diagnostic and public health microbiology laboratories. In this paper we described a system which is user-friendly, flexible and scalable, and which could be used by regular laboratory staff members, without specific bioinformatics training. This system overcomes the difficulties met by existing approaches, in regards to usability, scale and variety of genomic data which could be processed, data management, automation of required tasks, as well as auditing capabilities which are critical for any system in the public health sector.

## List of abbreviations used

NGS: Next Generation Sequencing, API: Application Programming Interface, MLST: Multi Locus Sequence Typing, ST: Sequence Type.

## Competing interests

The authors declare that they have no competing interests.

## Authors' contributions

LIR and KLW drafted the manuscript. CQ, TC, AB, LIR and SG work on developing the system described, in a prototype form. MR, DE, GH and KH were the initiators of the project and provided very useful feedback on the manuscript. All authors read and approved the final manuscript.

## Declarations

The publication charge for this article was funded by IBM Research - Australia.

This article has been published as part of *Health Information Science and Systems *Volume 3 Supplement 1, 2015: Proceedings of the Health Informatics Society of Australia Big Data Conference (HISA 2013). The full contents of the supplement are available online at http://www.hissjournal.com/supplements/3/S1/
